# Terpene arms race in the *Seiridium cardinale* – *Cupressus sempervirens* pathosystem

**DOI:** 10.1038/srep18954

**Published:** 2016-01-22

**Authors:** Ander Achotegui-Castells, Gianni Della Rocca, Joan Llusià, Roberto Danti, Sara Barberini, Mabrouk Bouneb, Sauro Simoni, Marco Michelozzi, Josep Peñuelas

**Affiliations:** 1 CREAF, Cerdanyola del Vallès 08193, Catalonia, Spain; 2 CSIC, Global Ecology Unit CREAF-CEAB-UAB, Cerdanyola del Vallès 08193, Catalonia, Spain; 3 IPSP-CNR, Via Madonna del Piano 10, I-50019, Sesto Fiorentino (FI), Italy; 4 CRA-ABP, Via Lanciola 12, Cascine del Riccio 50125 (FI), Italy; 5 IBBR-CNR, Via Madonna del Piano 10, I-50019, Sesto Fiorentino (FI), Italy

## Abstract

The canker-causing fungus *Seiridium cardinale* is the major threat to *Cupressus sempervirens* worldwide. We investigated the production of terpenes by canker-resistant and susceptible cypresses inoculated with *S. cardinale*, the effect of these terpenes on fungal growth, and the defensive biotransformation of the terpenes conducted by the fungus. All infected trees produced *de novo* terpenes and strongly induced terpenic responses, but the responses were stronger in the canker-resistant than the susceptible trees. *In vitro* tests for the inhibition of fungal growth indicated that the terpene concentrations of resistant trees were more inhibitory than those of susceptible trees. The highly induced and *de novo* terpenes exhibited substantial inhibition (more than a fungicide reference) and had a high concentration-dependent inhibition, whereas the most abundant terpenes had a low concentration-dependent inhibition. *S. cardinale* biotransformed three terpenes and was capable of detoxifying them even outside the fungal mycelium, in its immediate surrounding environment. Our results thus indicated that terpenes were key defences efficiently used by *C*. *sempervirens*, but also that *S. cardinale* is ready for the battle.

Terpenes, among the main defences of conifers, act as a first line of defence against biological agents and are usually strongly induced when trees are infected by bark-beetle/fungal pathogen complexes^1^,^2^. Terpene profiles are strongly genetically controlled, and conifers can differ greatly in their constitutive terpenes and defensive responses, depending on tree provenance, population, or variety^3^,^4^. Some studies have attempted to correlate terpenes with resistance in Pinaceae tree varieties against fungal pathogens, and even though links between pathogen resistance and increased terpene concentrations have been reported^5^,^6^, a consensus has not been reached due to other conflicting reports^4^,^7^. The ability of terpenes to inhibit spore germination and the growth of fungal pathogens is well known^8^,^9^. The inhibition caused by arbitrary concentrations of terpenes (especially monoterpenes (MTs)) has been tested on conifer pathogens, but experiments studying the effects of *in planta* concentrations are rare^10^,^11^. In the context of an arms race with trees, though, several specialised pathogenic fungi possess mechanisms of terpene biotransformation and detoxification^12^,^13^ and in some cases can even exploit these terpenes as carbon sources for their growth^14^,^15^. We still know little about terpenoid synthesis and biotransformation in fungi, with only three biotransformative pathways fully described genetically and enzimatically^16^. The biotransformation of terpenoids has been studied in only a few fungal pathogens of Pinaceae^12^,^17^, *Grosmannia clavigera* in particular^15^,^18^, so our understanding of fungal resistance to terpenes remains very poor, despite it is crucial to understand any conifer pathosystem.

*Seiridium cardinale* is the main agent of cypress canker, a severe pandemic disease reported for the first time 80 years ago, responsible for significant mortality in *Cupressus sempervirens* and most species of Cupressaceae worldwide^19^. The fungus is disseminated over short distances by airborne rainwater, and insect vectors may be responsible for its spread over longer distances^20^,^21^ (Fig. 1). The hyphae of *S. cardinale* infect the phloem, parenchyma, and cambium, occupying intercellular spaces and attacking cells with enzymes that degrade cell walls^22^. *S. cardinale* secretes several phytotoxins^23^, such as sesquiterpenes (STs) that cause systemic chlorosis and browning of leaves and uninfected plant tissues^24^,^25^. The phloem of infected canker-resistant trees produce *de novo* MTs and strongly induce several minor MTs and diterpenes (DTs)^26^, but information about non-resistant cypresses remains unavailable. Regarding fungal growth inhibition, only one study^27^ has tested *S. cardinale*, and found that two ST phytoalexins produced by *Diplodia pinea* f.sp. *cupressi*, (another canker-causing fungal pathogen) strongly inhibit its growth. To our knowledge, no other terpenes of *C. sempervirens* have been tested, and the terpene biotransformation capacity of this fungus has never been investigated. To fill these gaps in our understanding of the arms race between the tree and the fungus, we studied the terpenic composition and response of *C. sempervirens* trees selected for resistance against canker (Agrimed) and trees not selected for resistance (NR) to *S. cardinale* infection using gas chromatographic/mass spectrometric (GC-MS) analyses of control, wounded, and infected phloem tissues. We then used *in vitro* growth inhibition tests using both *in planta* and arbitrary concentrations to determine the antifungal activity of 15 terpenes in healthy and cankered *C. sempervirens*. We also studied the biotransformative and detoxificant capabilities of *S. cardinale* inside (hyphae, H) and outside (hyphae-free, HF) the mycelium with GC-MS analyses of *in vitro* inhibition test plugs.

## Results

**Figure 2:**
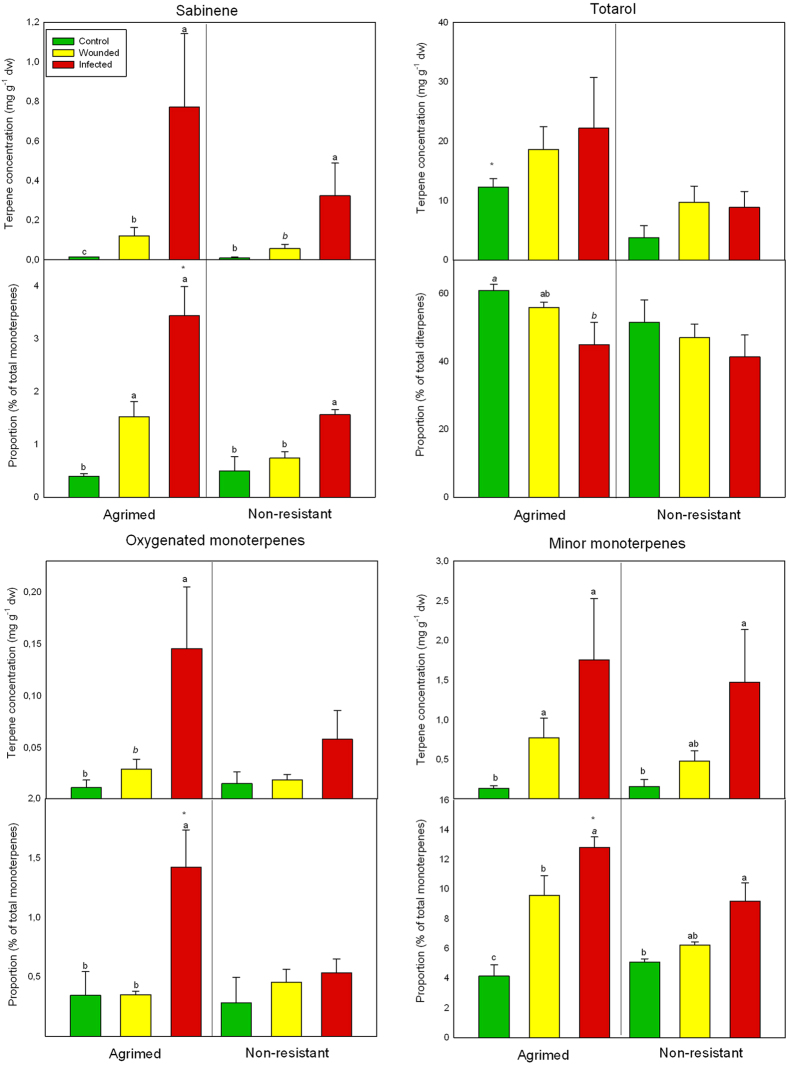
Mean phloem concentrations (mg g^−1^ dry weight ± SE) and proportions (% relative to the terpene class ± SE) of sabinene, totarol, oxygenated monoterpenes, and minor monoterpenes for the two *Cupressus sempervirens* groups, Agrimed (canker resistant) and non-resistant (NR). Different letters indicate significant differences among treatments of the same group (one-way ANOVA, Tukey’s *post hoc* test, *P* < 0.05). Asterisks (*) indicate significant differences (one-way ANOVA, Tukey’s *post hoc* test, *P* < 0.05) between the same treatments of both groups. dw, dry weight.

Terpenic composition differed substantially between tree groups and treatments 30 days after artificial inoculation. Sabinene hydrate, camphor, and oxygenated MT1 and 2, were *de novo* terpenes exclusively found in the infected states of both groups (oxygenated MT2 only in infected Agrimed). Other compounds, such as ocimene, thymyl methyl eter, and MT4 were only found in the wounded and infected states. The concentrations of these terpenes were usually low (Table 1). DTs were the main fraction (70–80% of total terpenes, led by totarol) in the phloem of both cypress groups, followed by MTs (20–30%, led by α-pinene and δ-3-carene) and STs (ca. 1%, led by cedrol) (Table 1). The concentrations of terpenes in both cypress groups, especially MTs and DTs, tended to be higher in the infected than the wounded and control treatments (one-way ANOVA, Tukey’s *post hoc* test *P* < 0.05 or *P* < 0.10) (Fig. 2, Table 1). Infected Agrimed had higher concentrations than the wounded or control treatments (of 16 terpenes) more often than infected NR (of eight terpenes) (Table 1). Agrimed had higher concentrations than NR of longifolene, totarol, and total DTs in the control treatments and of ocimene in the infected treatments (Table 1, Fig. 2). The proportions of terpenes (relative to their class) followed similar trends but also decreased for some compounds, especially the most abundant terpenes (Table 1, Fig. 2). Agrimed was again more responsive to infection, with 25 terpenes significantly changing proportions (19 increases and six decreases) than NR, with 11 changes (10 increases and one decrease). Infected and wounded Agrimed had higher proportions than NR of 20 terpenes, mostly MTs.

**Figure 3:**
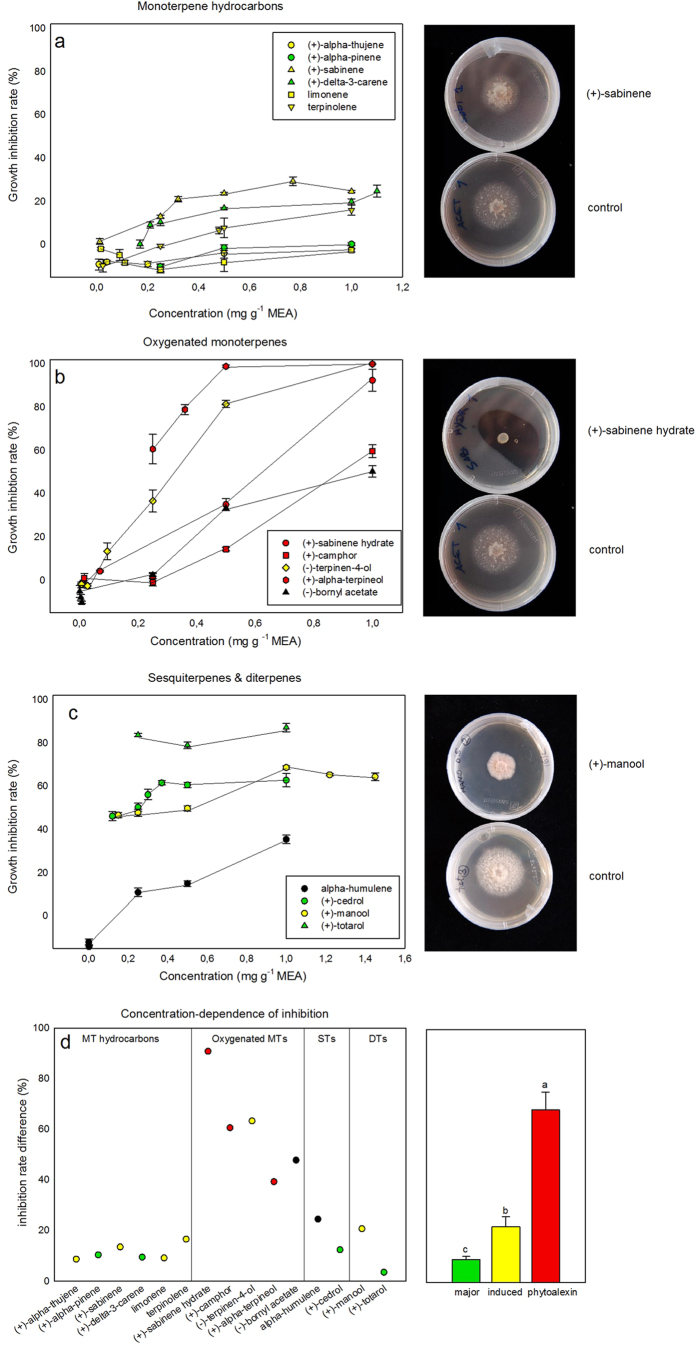
Inhibition-rate curves of fungal growth (mean ± SE) and photographs of growth inhibition for **a)** monoterpene hydrocarbons, **b)** oxygenated monoterpenes, and **c)** sesquiterpenes and diterpenes, and d) the results of a concentration-dependence test (difference between the inhibitions of the 1.0 and 0.25 mg g^−1^ MEA tests). Different letters in the histogram in d) indicate significant differences (one-way ANOVA, Tukey’s *post hoc* test, *P* < 0.05). Green, main terpenes of each terpene class; yellow, canker-induced terpenes; red, phytoalexins; black, uncategorised. Photograph credit: Gianni Della Rocca.

The antifungal activity against *S. cardinale* of the *C. sempervirens* terpenes varied substantially when tested *in vitro*, ranging from complete growth inhibition (e.g. (+)-α-terpineol and (−)-terpinen-4-ol) to growth stimulation (e.g. (+)-α-pinene and limonene) (Fig. 3, Table 2). Inhibition appeared to be concentration-dependent for most terpenes, with several concentration-inhibition patterns (Fig. 3). Several of the simulated concentrations in the *in planta* tests showed different inhibition power among the control, infected Agrimed, and infected NR (one-way ANOVA, Tukey’s *post hoc* test, *P* < 0.05) (Table 2, Fig. 3). The *in planta* concentrations of infected Agrimed were more inhibitory than the control for all compounds except (+)-α-pinene, (−)-bornyl acetate and limonene. Infected NR concentrations were more inhibitory than the control concentrations for (+)-sabinene, terpinolene, (+)-cedrol and (+)-manool, and infected Agrimed concentrations were more inhibitory than infected NR concentrations for (+)-sabinene, (+)-δ-3-carene, and (−)-terpinen-4-ol. The mean of all inhibitions of infected Agrimed (24.1%) was significantly higher than that of infected NR (18.4%) and the control (15.0%) (one-way ANOVA, Tukey’s *post hoc* test, *P* < 0.01).

Only some oxygenated MTs, (+)-cedrol, and the DTs had substantial effects on fungal growth in the fixed concentration tests (Table 2) at 0.25 mg g^−1^ malt agar extract (MEA). (+)-Totarol was more inhibitory than azoxystrobin, a reference fungicide. At 0.50 mg g^−1^ MEA, some MT hydrocarbons began to show moderate rates of inhibition (ca. 25%), the oxygenated MTs substantially increased their inhibition, whereas STs and DTs maintained similar inhibitions to growth. The most concentrated test, 1.0 mg g^−1^ MEA, exhibited the strongest inhibitions, led by oxygenated MTs, half of which inhibited growth completely and overcame the inhibition caused by the fungicide, followed by DTs, STs, and MTs. We calculated the concentration-dependence of inhibition for each terpene within that concentration range (Fig. 3d) by subtracting the inhibition in the 0.25 mg g^−1^ MEA test from the inhibition in the 1.0 mg g^−1^ MEA test. Oxygenated MTs were the most concentration-dependent class of terpenes compared to MT hydrocarbons, STs and DTs (one-way ANOVA, Tukey’s *post hoc* test, *P* < 0.05). The most concentration-dependent compounds were the *de novo* terpenes (68.2%), followed by induced terpenes (22.1%), and the major terpenes (9.0%) (one-way ANOVA, Tukey’s *post hoc* test, *P* < 0.01) (Fig. 3d).

**Figure 4:**
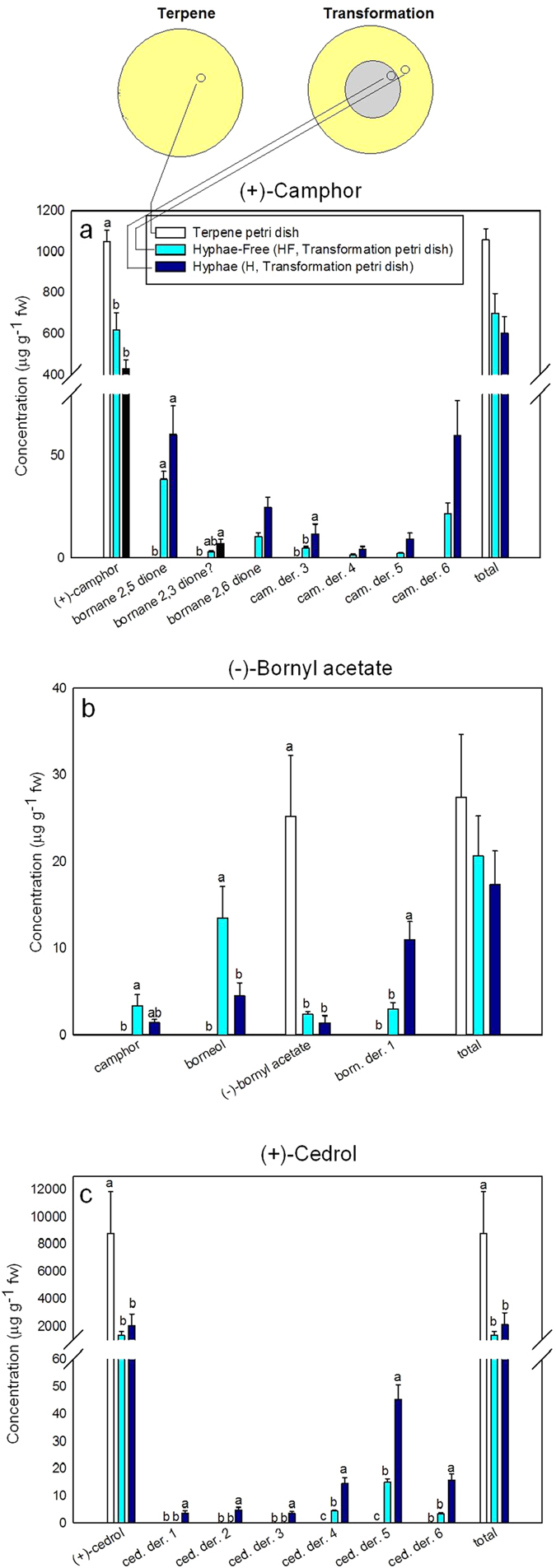
Mean concentrations (mg g^−1^ fresh weight ± SE) of compounds in the plugs of malt agar extract (MEA) extracted from tests performed with **a)** (+)-camphor, **b)** (−)-bornyl acetate and **c)** (+)-cedrol in the terpene (MEA + acetone + terpene) and transformation (MEA + acetone + terpene + fungus) tests. Different letters indicate significant differences (one-way ANOVA, Tukey’s post hoc test, P < 0.05). fw, fresh weight; cam. der., (+)-camphor derivative; born. der., (−)-bornyl acetate derivative; ced. der., (+)-cedrol derivative. ?, tentative identification.

**Figure 5:**
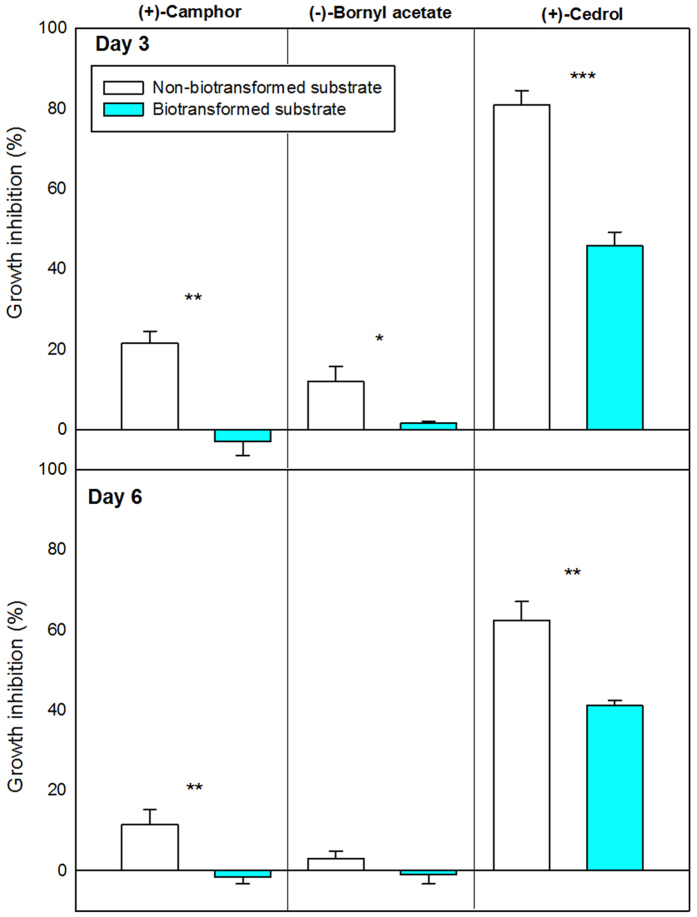
Fungal growth inhibition (mean ± SE) measured at day 3 (top) and day 6 (bottom) provoked by the the application of pentane extracts of Non-biotransformed substrate and Biotransformed substrate. Asterisks (*) indicate statistically significant differences between the two treatments (T-tests, *P < 0.05, **P < 0.01, ***P < 0.001). Bar colors are in accordance with those of Fig. 4.

Several biotransformations in both H (hyphae) and HF (hyphae free, 0.5 cm away from the mycelial border) plugs were detected in the biotransformation tests where *S. cardinale* grew on MEA plates containing (+)-camphor, (−)-bornyl acetate, or (+)-cedrol (the transformation test), but only the terpene substrate was found on MEA plates containing these three terpenes but without the fungus (the terpene test) (Fig. 4). The Petri dishes with fungus grown on a substrate of (+)-camphor had six new compounds, three of which were identified as bornane-2,5-dione, bornane-2,3-dione (tentative identification), and bornane-2,6-dione. Fungus grown on MEA containing (−)-bornyl acetate generated three biotransformation products, two of which were identified as camphor and borneol. *S. cardinale* grown on (+)-cedrol produced six new compounds that could not be identified. The tests also produced quantitative differences among these three terpenes (Fig. 4), and in all cases, the terpene substrate concentrations were higher in the MEA from the plates of the terpene test than H and HF of the transformation test (one-way ANOVA, Tukey’s *post hoc* test, *P* < 0.01). The H and HF samples of the transformation test also presented several differences, with H usually having higher concentrations of biotransformation products than HF (Fig. 4). A test to assess detoxification (Fig. 5) showed how the HF substrate of the three biotransformed terpenes ((+)-camphor, (−)-bornyl acetate and (+)-cedrol) was significantly less inhibitive to fungal growth than the HF substrate of non-biotransformed terpenes (T-tests *P* < 0.05).

## Discussion

Agrimed responded more strongly to infection than NR, producing an extra *de novo* oxygenated MT and more inductions in concentrations and proportions. Agrimed also had several higher concentrations and proportions of various terpenes than NR in the infected treatments (Table 1). Our results thus agreed with those from studies that correlated increased terpene concentration with infection resistance in conifers^5^,^6^,^28^. The current results (branch inoculations) agreed with those of our previous study^26^ (stem inoculations), despite some differences likely associated with the different phloem samples analysed^29^,^30^. A comparison of both studies suggests that branches, despite exhibiting a similar response, are less protected than the trunk, supporting field observations that found most of the cankers initiate in the axils of young branches^19^. The terpenes found in Italian cypress tissues in response to *S. cardinale* infection may not only be produced by the tree, as endophytic microorganisms could be contributing to cypress defence^31^. However, it is technically very difficult to separate the real effect of those microorganisms from the ‘pure’ response of the plant. Further research should try to ascertain the contribution of endophytes to *C. sempervirens* terpene defence against *S. cardinale*.

The majority of terpenes showed a concentration-dependent inhibition of fungal growth^12^,^32^ (Fig. 3). Concentration thus determined the ultimate capacity of inhibition (*in planta* tests, Table 2, Fig. 3), despite different inhibitions for some terpenes at equal concentrations (fixed tests). Agrimed responded to infection stronger than NR, and its concentrations also appeared to be more inhibitory to fungal growth in the *in planta* tests. Differences in inhibition between the concentrations of both infected groups occurred only for the MTs ((+)-sabinene, (+)-δ-3-carene, and (−)-terpinen-4-ol), so these results suggest that, by day 30, MTs could be the class of terpenes responsible for conferring the higher canker resistance to Agrimed (Table 2). The low concentrations of the oxygenated MTs (except terpinen-4-ol) and the low dependence of inhibition on the concentration of STs and DTs prevented these terpenes from causing significantly different inhibitions between groups by day 30, despite reports of being strong inhibitors of fungal growth^33^,^34^. The lack of antifungal activity reported for (+)-camphor and (+)-sabinene hydrate in the *in planta* tests could be due to their low concentrations^26^ and/or the detoxification capacity of *S. cardinale*. However, these low phytoalexin concentrations contrast with other reports^27^ where other antifungal phytoalexins produced by the infected stem of *C. sempervirens* were induced from day 2 (in our case we detect them from day 30 on) in concentrations about 1–2 mg g^−1^ (fresh weight). As said above, *S. cardinale* began to infect cypress a few decades ago, and we hypothesize that a lack of co-evolution could explain our results.

Despite being a useful tool for studying inhibition more realistically, *in planta* inhibition tests have two important limitations: *i*) mean concentrations in phloems are applied, which does not represent the real variability of concentrations, and *ii*) the application of the same concentrations in the MEA as those found in phloems may not be quantitatively appropriate. Our results suggested that X mg g^−1^ MEA were more inhibitory than X mg g^−1^ phloem. The fixed concentration tests allowed a comparison of the inhibitory powers of the terpenes and can help to predict inhibition in canker-infected cypress stems or more advanced states of infection (e.g. day 90), which should exhibit higher concentrations^26^ than those in the current study.

Our results suggest that the low inhibitory power of MT hydrocarbons is likely due to their high volatility and widespread occurrence in nature. In addition, several studies have reported that some terpenes, usually the most abundant compounds of a host, can enhance the growth of pathogens of conifers^35^,^36^. In our study, the oxygenated MTs, well-known inhibitors of fungal growth^8^,^12^, were the most inhibitory compounds at high concentrations. The *de novo terpenes* (+)-sabinene hydrate^37^, (+)-camphor^38^,^39^, and (+)-α-terpineol^12^,^40^, known to exhibit antifungal activity, were among the most inhibitory compounds in the fixed concentration tests and thus should be considered as phytoalexins against *S. cardinale*. The *de novo* compounds produced by an infected conifer can have very strong inhibitory effects on the infecting pathogen^27^,^35^. The oxygenated MTs had low inhibitory rates at the *in planta* concentrations but would likely have been stronger inhibitors at the ca. fifteen-fold higher concentrations reported in our previous study^26^, as suggested by their concentration-inhibition curves (Fig. 3) and the (+)-α-terpineol test (Table 2). STs represented only ca. 1% of the total terpene concentration in our study, but (+)-cedrol, the main ST, can be very inhibitory to fungi^34^ and maintained high rates of inhibition (ca. 60%) even at low concentrations. DTs also had strong inhibitory power, even at low concentrations. Constitutive totarol (higher in Agrimed (Fig. 2)) could be an effective first line of defence against fungal infection (Fig. 3). (+)-Totarol can inhibit efflux-pump activity in bacteria^41^, which could be related to its low concentration-dependent inhibition of *S.cardinale* (Fig. 3). The level of inhibition by the major terpenes of *C. sempervirens*, ((+)-α-pinene, (+)-δ-3-carene, (+)-cedrol, and (+)-totarol), differed little between 0.25 and 1.0 mg g^−1^ MEA (Fig. 3d), suggesting that their inhibitory capacities have a low dependence on concentration within this concentration range. In contrast, inhibition by oxygenated MTs (containing all three phytoalexins and the strongly induced terpene terpinen-4-ol) was very concentration-dependent (Fig. 3), perhaps accounting for the higher concentrations of several minor compounds such as the oxygenated MTs (Fig. 2), terpinolene, or manool rather than of major compounds. Differences in the concentrations and proportions of several specific terpenes between groups may partly account for the ability of Agrimed to resist cypress bark canker, which develops further in NR (eventually resulting in death). Our results thus support the hypotheses proposed in our previous study^26^, which suggested that terpenes may function to slow fungal advance, enhance compartmentalisation by necrophylactic periderm, and ultimately stop the fungal infection. In this work we have studied the role of terpenes in cypress defense against *S. cardinale* infection, but nothing is known about the defensive role of other plant secondary metabolites such as phenols. Further investigations should study, with e.g. high resolution MS, the defensive reactions of these compounds in order to better understand role and importance of each group of secondary metabolites in cypress defence.

The biotransformation of (+)-camphor to bornane-2,5-dione was observed for the first time in *Pseudomonas putida*^42^, and this biotransformative pathway has since been extensively studied, mainly in bacteria^43^,^44^. The fungal biotransformation of bornyl acetate to camphor and borneol has also been described^12^,^45^. Detoxification was observed (Fig. 5) in HF biotransformed substrate for (+)-camphor, (−)-bornyl acetate and (+)-cedrol, suggesting that *S. cardinale* is capable of detoxifying^46^ its immediate environment on its behalf. The biotransformations and detoxifications observed in the MEA free of fungal hyphae (HF) could be explained by two processes: *i) S. cardinale* excretes terpene substrates along with some biotransformed products away from fungal cells, which would act as a detoxification mechanism to lower the cellular terpene levels^15^. This explanation, though, is inconsistent with the significantly lower concentrations of terpene substrates in the HF plugs of the transformation tests relative to those of the terpene tests (Fig. 4). *ii) S. cardinale*, suggested to release exoenzymes that play a role in systemic pathogenesis^47^, may also have secreted exoenzymes capable of degrading defensive terpenes before hyphal contact. Such a mechanism would be advantageous to *S. cardinale*, because the fungus would encounter partially detoxified defences, and thus a less aggressive environment to colonise.

## Conclusions

The differences in constitutive and induced terpene responses to infection between NR and Agrimed, along with the inhibitory power of these compounds, suggest that part of the Agrimed resistance to cypress bark canker may be due to its stronger and more inducible terpenic profile. Inhibition tests suggest that *C. sempervirens* reacts to the early stages of infection (day 30) by increasing the concentration of MTs but may be preparing itself for more advanced stages by beginning to generate several phytoalexins and increasing the concentrations of the most inhibitory compounds currently known for this pathogen. Cypress devoted more resources to increasing the concentrations of minor than of major terpenes, corroborating the observations of our previous study^26^, and we suggest that this strategy may be due to the high concentration-dependent inhibition of the most highly induced minor terpenes and the low concentration-dependent inhibition of the major terpenes. Nevertheless, *S. cardinale* may be able to tolerate some of the most inhibitory terpenes of *C. sempervirens*, detoxifying them by biotransformation and changing its immediate environment for its behalf. Further studies should determine the identity of the biotransformed compounds, and investigate the biotransformation and detoxification mechanisms of this fungus. It would also be very interesting to see if other pathosystems react similarly and corroborate the tree defence mechanisms suggested here. In more practical terms, the strong actions of the most inhibitory terpenes warrant further efforts to test their viability as natural fungicides against *S. cardinale*.

## Methods

### Terpene concentrations

#### Plant and fungal material

Thirty-six five-year-old grafted *Cupressus sempervirens* L. trees grown in pots were divided into two groups: 18 were not selected for resistance to cypress bark canker (NR) and 18 were the Agrimed n°1^48^ cultivar (hereafter Agrimed) patented for canker resistance. The plants were maintained under a shedding tunnel at ISZA-CRA in Firenze (Italy) and were watered daily. The *S. cardinale* (Wagener) Sutton & Gibson standard isolate ATCC 38654 was used for the artificial inoculations and the inhibition and biotransformation tests. The fungus was grown on malt agar extract (MEA) at 25 °C in the dark for 15 days.

#### Inoculation and sampling

Three treatments were applied to both tree groups in August 2013: control (no inoculation wound, no fungus), wounded (inoculation wound, no fungus), and infected (inoculation wound + fungus). The phloems of three randomly chosen main branches of the trees were inoculated following standard procedures^26^. Each treatment had six replicates, and each replicate consisted of three sub-replicates (three branches). Phloem tissues were sampled 30 days after inoculation, kept in liquid nitrogen and stored in a −20 °C freezer.

#### Sample analyses and terpene identification

The phloem sub-samples of each replicate were bulked and ground with a pestle in 50-ml Teflon tubes containing liquid nitrogen to avoid evaporation and facilitate the grinding. One ml of pentane containing dodecane (internal standard) was added to the ground tissues, and the solution was stored overnight at −20 °C. Three-hundred μl of the supernatant were analysed by GC-MS. The Teflon tubes were dried to constant weights, weighed in a precision balance, cleaned thoroughly, dried, and reweighed to tare the tubes. One blank was analysed for every six samples.

Two microlitres of the phloem extract were injected into a capillary column (HP 5MS, 30 m × 0.25 μm × 0.25 mm) of a GC (7890A, Agilent Technologies, Santa Clara, USA) with an MS detector (5975C inert MSD with Triple-Axis Detector, Agilent Technologies). Initial temperature was maintained at 35 °C for two minutes, increased at 15 °C min^−1^ to 150 °C and maintained for 5 min, thereafter at 30 °C min^−1^ to 250 °C and maintained for 5 min, and finally at 30 °C min^−1^ to 280 °C and maintained for 5 min. Total run time was 29 min, the helium flow was set to 1 ml min^−1^, and the split was 1:10. The terpenes were identified by comparing the mass spectra with known standards and published spectra (NIST 05, NIST 08, and Wiley 7n libraries). Calibration curves for terpene quantification were prepared with dodecane and commercial standards of four MTs (α-pinene, sabinene, δ-3-carene and γ-terpinene), four STs (caryophyllene, caryophylene oxide, cedrol and farnesol) and two DTs (totarol and phytol). All terpenes were purchased from Fluka Chemie AG, Buchs, Switzerland, and had purities superior to 95%. Four different concentrations were used to perform terpene calibration curves, with correlations between signal and concentration always highly significant (*r*^2^ ≥ 0.99). The main terpenes had similar sensitivities (differences < 5%).

### Antifungal assays

The terpenes tested for antifungal activity were selected by their performance in our previous study^26^, the current study, and compound chirality observations (Michelozzi, unpublished results). The tested terpenes were: (+)-α-thujene, (+)-α-pinene, (+)-sabinene, (+)-δ-3-carene, limonene (unknown chirality), terpinolene (unknown chirality), (+)-sabinene hydrate, (+)-camphor, (−)-terpinen-4-ol, (+)-α-terpineol, (−)-bornyl acetate, α-humulene (unknown chirality), (+)-cedrol, (+)-manool, and (+)-totarol. All compounds had purities superior to 95%, except (+)-α-thujene and terpinolene (90% of purity) and were obtained from Fluka Chemie AG, (Buchs, Switzerland), except (+)-α-thujene (Chemos GmbH, Regenstauf, Germany) and (+)-manool (Sequoia Research Products Limited, Pangbourne, UK). We used the broad-spectrum fungicide azoxystrobin (Quadris^®^, Syngenta), commonly used against cypress bark canker^49^, as a positive control in the antifungal tests.

The *in vitro* tests were performed in 6-cm Petri dishes containing 5 g of 2% MEA. The test solutions were prepared by mixing the terpenes with 60 μl of acetone, whereas the acetone controls contained only acetone. The solutions were gently shaken, pipetted, and then spread over the MEA surface with a spatula. A 5-mm disk of a *S. cardinale* colony was then placed in the centre of the Petri dishes, which were immediately tightly sealed with Parafilm^®^ and incubated at 25 °C for 6 d in the dark. All tests were replicated four to five times. Three *in planta* concentrations corresponding to those found in the *C. sempervirens* GC-MS (in mg g^−1^ phloem tissue) analyses were tested for each terpene *in vitro* (in mg g^−1^ MEA). The control (mean concentration of both groups of trees), infected NR, and infected Agrimed (Table 2) concentrations were tested. Three arbitrarily fixed concentrations were tested to compare the inhibitory powers of the terpenes: 0.25, 0.50, and 1.0 mg g^−1^ MEA. Two perpendicular diameters of the fungal colonies were measured after 3 d and 6 d. Growth-inhibition rates (%) were calculated by:


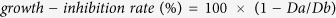


where *Da* is the average mycelial diameter of each sample test and *Db* is the average mycelial diameter of the acetone control.

### Biotransformation

MEA plugs (5 mm diameter) from the 1.0 mg g^−1^ MEA inhibition tests (transformation, MEA + acetone + terpene + fungus) were sampled immediately after the measurement of fungal growth at day 6 to study fungal biotransformations. Three supplementary tests, agar (MEA + acetone, to detect MEA metabolites), fungus (MEA + acetone + fungus, to detect fungal metabolites), and terpene (MEA + acetone + terpene, to detect terpene oxidations or degradations), were also sampled 6 d after test application. No terpenes were detected in the agar and fungus tests. Three plugs were taken from the transformation test, one from the margin of the colony (hyphae, H), one of MEA 5 mm from the mycelial border (hyphae-free, HF), and one identical to HF to incubate for two weeks for verifying the absence of hyphae (no *S. cardinale* grew in the HF plugs for verifying the absence of hyphae). Plugs were extracted with a 5-mm cork borer and were placed in refrigerated Eppendorf tubes and immediately stored at −80 °C. Sample analyses and identification were performed as described above but with individual calibration curves for each terpene and a split of 1:3 to maximise compound detection. The GC-MS analyses of the MEA found no MTs^12^, but most of the oxygenated MTs, STs, and DTs had consistent concentrations.

### Detoxification

The inhibitory activity of the terpenes biotransformed by *S. cardinale* on the fungus mycelial growth was assessed *in vitro*. Two groups (detoxification and control) of 12 Petri dishes containing MEA and 1 mg g^−1^ of (+)-camphor, or (−)-bornyl acetate or (+)-cedrol (commercial standards) solved in pentane (4 replicates for each terpene) were prepared following the procedure described before. In each Petri dish of the first group (detoxification treatment), a 5-mm plug of *S. cardinale* mycelium was added as described above. No *S. cardinale* plug was added to the second group of Petri dishes (control treatment). The Petri dishes were stored at the previously described conditions for 6 days.

Then, a 10 mm width ring of MEA+terpene (hyphae free) surrounding the *S. cardinale* colony was trimmed from each Petri of the detoxification treatment. In the control Petri dishes, we trimmed the equivalent ring of agar from an equivalent position. The trimmed rings of MEA from each Petri dish were separately soaked with 3 ml of pentane in hermetic vials and kept overnight at −20 °C in constant shaking (150 rpm) in order to extract all the terpenes and the products biotransformed by the fungus. Next morning, we concentrated the resulting extract with a flux of gaseous nitrogen until we reached a final volume of 100 μl. This final solution was pipetted and spread in the surface of two new groups of MEA Petri dishes (4 replicates each): Biotransformed substrate (extract of MEA with biotransformation products of *S. cardinale*) and Non-biotransformed substrate (extract of MEA with non-biotrasformed terpenes). Petri dishes were incubated as previously described conditions and fungal growth was measured after 3 and 6 days.

### Statistical analyses

The data were analysed by one-way ANOVAs, and treatments and tests were compared with Tukey’s *post hoc* tests (*P* < 0.05). Outliers were removed using absolute deviation around the median^50^. Data not fitting the requirements of normality were transformed or analysed with non-parametric methods (Kruskal-Wallis one-way ANOVA). The statistical analyses used Statistica version 8.0 (StatSoft Inc. Tulsa, USA), SigmaPlot version 11.0 (Systat Software, Chicago, USA), and R software version 2.15.2 (R foundation for Statistical Computing, 2012).

## Additional Information

**How to cite this article**: Achotegui-Castells, A. *et al.* Terpene arms race in the *Seiridium cardinale - Cupressus sempervirens* pathosystem. *Sci. Rep.* **6**, 18954; doi: 10.1038/srep18954 (2016).

## Figures and Tables

**Figure 1:**
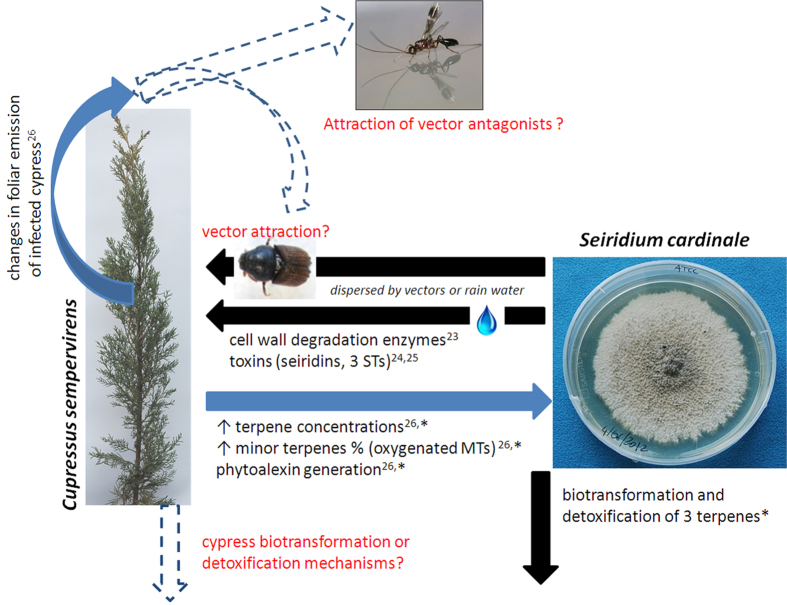
Scheme depicting the interactions between host and pathogen in which terpenes play or may play a role. Black arrows indicate fungal activity, blue arrows indicate tree activity, and dashed arrows with red labels indicate possible but yet unknown interactions. Asterisks (*) indicate the findings of the current study. Photograph credit: All photographies taken by Gianni Della Rocca, except “antagonist” (USDA).URL: https://en.wikipedia.org/wiki/Spathius_agrili#/media/File:Spathius_agrili.png

**Table 1:**
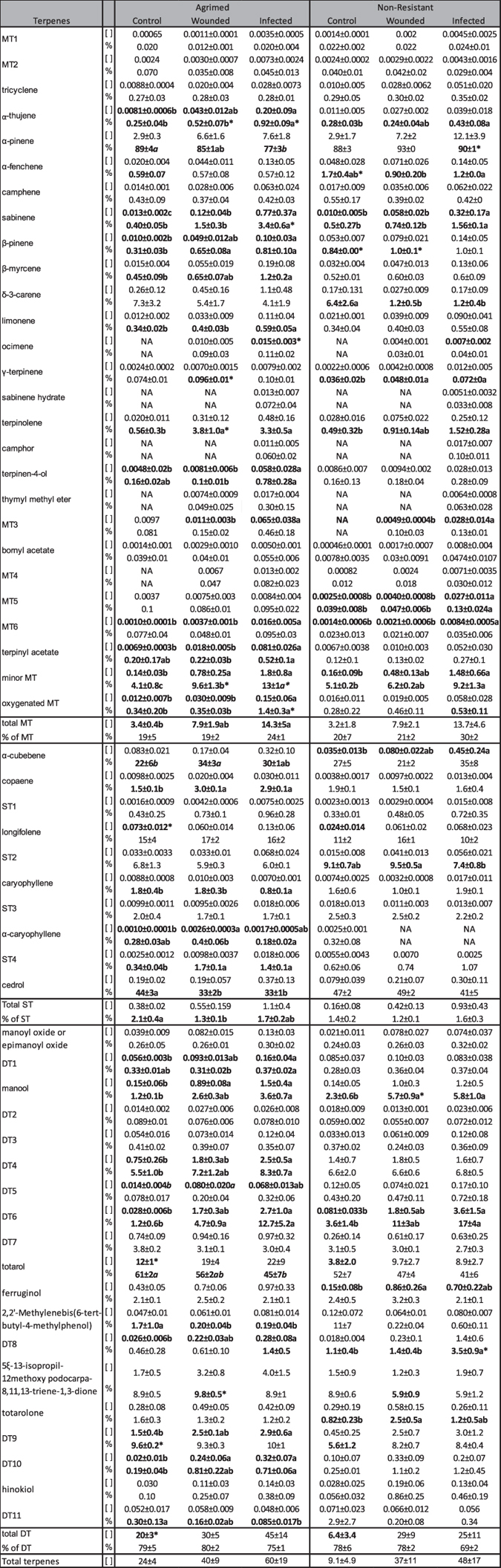
Mean concentrations (mg g^−1^ dry weight ±SE) and proportions (% ±SE) relative to the class of the terpenes in the local phloem of canker-resistant (Agrimed) and non-resistant (NR) cypresses.

Numbers and letters in bold type indicate significant (one-way ANOVA, Tukey’s *post hoc* test, *P* < 0.05) and marginally significant (*P* < 0.10, in *italics*) differences within the treatments of a tree group. Asterisks (*) indicate statistically significant differences between the same treatment of different groups (one-way ANOVA, Tukey’s *post hoc* test, *P* < 0.05). [ ], concentration; MT, monoterpene; ST, sesquiterpene; DT, diterpene; NA, not available.

**Table 2:**
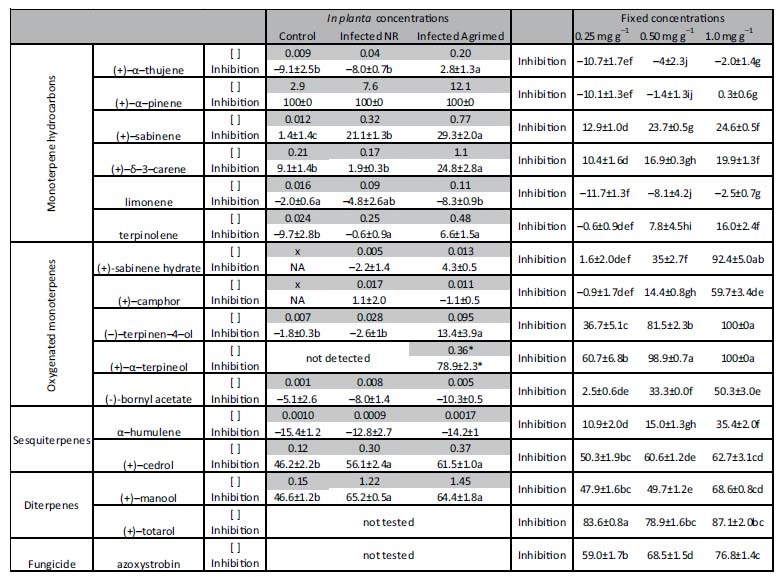
Mean rates of growth inhibition (% ±SE) of *Seiridium cardinale* by 15 terpenes (11 monoterpenes, two sesquiterpenes, and two diterpenes) and one fungicide relative to the inhibition by acetone (control).

The *in planta* concentrations tested the same terpene concentrations as those in the phloem (Table 1) in the different treatments (control, infected NR, and infected Agrimed) applied per gram of malt extract agar (MEA). The fixed concentrations tested three arbitrary concentrations (0.25, 0.50, and 1.0 mg g^−1^ MEA) for comparing the inhibitory power among several terpenes. Different letters indicate significant (one-way ANOVA, Tukey’s *post hoc* test, *P* < 0.05) and marginally significant (*P* < 0.10, in *italics*) differences within the treatments of a tree group. Comparisons for the *in planta* concentration tests were performed between treatments (horizontal), and comparisons for the fixed concentrations were performed within the treatments (vertical). The asterisks (*) for (+)-α-terpineol indicate that this test was performed with the concentration found in a previous study^26^, because we did not detect this compound in the current study.
